# Investigation of p16 protein expression and its association with histopathologic parameters in breast cancer

**DOI:** 10.22099/mbrc.2021.41691.1671

**Published:** 2021-12

**Authors:** Siamak Naji-Haddadi, Daniel Elieh-Ali-Komi, Saeid Aghayan, Rahim Asghari, Javad Rasouli

**Affiliations:** 1 Mahzad Women’s Hospital Urmia Medical Sciences University (UMSU), Urmia, Iran; 2 Cellular and Molecular Research Center, Cellular and Molecular Medicine Institute, Urmia University of Medical Sciences, Urmia, Iran; 3 Department of Pathology, Urmia University of Medical Sciences, Urmia, Iran; 4 Immune Cell Therapy, Stem Cell Transplantation Research Center (HİSRC), Urmia Medical University (UMSU), Urmia, Iran; 5 Department of Biostatistics and Epidemiology, School of Medicine Urmia University of Medical Sciences, Urmia, Iran; ¥ Siamak Naji-Haddadi and Daniel Elieh-Ali-Komi contributed equally to the project.

**Keywords:** Breast cancer, p16, Neural invasion, Vascular invasion, Nodal involvement

## Abstract

We investigated the association between p16 expression and histopathologic parameters including size, neural and vascular invasion, and lymph node involvement in breast cancer. 58 specimens from patients with different grades of breast cancer were included. Hematoxylin and eosin and immunohistochemistry staining for p16 was performed. 5 patients (8.6%) had grade I, 23 (39.7%) had grade II, and 30 (51.7%) had grade III breast cancer. Assessment of the tumor size showed that 5 (8.6%) tumors had a size of ≤2cm, 29 (50%) were between 2-5 cm and 24 (41.4%) had a size of ≥5cm. Moreover, 45 (77.6%) of the included patients had axillary lymph node involvement. Investigation of association between p16 positivity with pathological parameters in three groups with positivity to p16 (1-25%, 26-75%, >75%) showed that there was no association between p16 positivity and other parameters including histologic score (p=0.44), tumor size (p=0.77), neural invasion (p=0.79), perivascular invasion (p=0.98) and the number of involved LNs (p=0.49). From the group including eight patients with >75% p16 positivity, seven (87.5%) were found with neural invasion and two (25%) with perivascular invasion. P16 positivity was not associated with size, neural and vascular invasion, and LN involvement in breast cancer.

## INTRODUCTION

 Breast cancer (BC) is the most commonly diagnosed cancer in Iranian women and its mortality rate is an alarming trend [1-3]. The main BC subtypes include luminal A, luminal B, HER2-overexpressing, and triple negative that vary in terms of gene expression, prognosis, and responsiveness to chemotherapy [4]. Investigation of estrogen receptor (ER), progesterone receptor (PGR), and epidermal growth factor receptor-2 (HER2) expression is a common approach to classify the subtypes using immunohistochemistry (IHC) [5, 6]. Genetic mutations (inherited and acquired) and epigenetic aberrations are the most crucial contributors to the susceptibility of women to BC [7]. 

p16 is a nuclear protein that is encoded by the p16INK4a gene and acts as a negative regulator of cell proliferation. The mechanism of action includes inhibition of the phosphorylation of retinoblastoma (RB) family members through binding to CDK4/6 [8]. p16/RB regulates G1 to S transition in cell cycle [9]. Inactivation of the pathway has been reported in human cancers such as hepatocellular carcinomas [9] and non-small-cell lung cancers [10]. Additionally, hypermethylation and mutation in P16 gene contribute to the progression of cancer [11]. However, inconsistent results regarding the biofunction of p16 in BC have been reported. In this regard, it has been reported that inhibition of P16 could decrease the growth and metastasis potential of BC cells through inhibiting IL-6/JAK2/STAT3 signaling and that the inhibition of P16 could result in decreasing the tumor growth rate in *in vivo* BC xenograft models [12]. Most recently, the investigation of BC-related marker expression provided valuable results even when different types or stages of BC were studied. For example, a COX-2+p16+Ki67+ phenotype was found dominant in premalignant lesions [13]. We investigated the expression of p16 protein and its association with histopathologic parameters including size, neural and vascular invasion, and lymph node (LN) involvement in 58 breast cancer samples with different histopathologic grades.

## MATERIALS AND METHODS

**Patients and collection of BC specimens: **BC specimens were collected from 58 BC patients hospitalized in Urmia medical university hospital, Iran. The mean age was 51.7± 11 (min= 29, max= 79, median=51). 4μm sections were prepared from formalin-fixed, paraffin-embedded blocks. All included patients met the following criteria: (1) diagnosed with primary BC; (2) having complete clinicopathological records and follow-up information. 

This study was approved by the ethics committee at Urmia Medical University for screening, inspection, specimen collection, and analyzing data. All included patients signed the consent form and all the procedures were performed in accordance with the Declaration of Helsinki.

**P16 immunohistochemistry testing and tumor grading: **In this study, we included 58 patients with BC who had undergone tumor resection. We performed hematoxylin and eosin (H&E) staining on specimens and the appropriate blocks were selected for IHC staining. 4μm sections were prepared from the blocks and IHC staining for p16 expression was performed. Grading was performed by the Bloom–Richardson method [14], and staging according to TNM(AJCC) system (**T**: the size of the tumor **N:** the spread to nearby LNs, and **M:** the spread (metastasis) to distant sites). The histopathologic features, invasiveness, and LN involvement were studied. After IHC staining the p16 positivity was reported on the scale of 0-3 in which 0 represented the negative p16 staining, 1 represented p16 positivity of 1-25%, 2 represented P16 positivity of 26-75%, and >75% P16 positivity was represented as 3. To eliminate any possible bias, we used a masking system to report the results of IHC staining in which all slides were numbered and the pathologists were not aware of the identification or medical history of the patients.

**Statistical analyses:** SPSS16 (IBM-USA) was used for analyzing data. We used frequency-distribution tables to report descriptive statistics, and Chi-squared test to compare them. Data was represented as mean ± standard deviation (SD), and differences between groups were compared using student's t-test and ANOVA. A p<0.05 was considered to be statistically significant.

## RESULTS

The histologic grading of specimens was performed according to Nottingham modification of the Scarff-Bloom-Richardson (NSBR) histological grading system. Five patients (8.6%) had grade I, 23 (39.7%) had grade II, and 30 (51.7%) had grade III BC. The maximum and minimum size of tumors were 10 and 0.8 cm respectively (mean tumor size 4.91± 2.05 cm). According to the size of the tumors, we placed them in three groups with different tumor sizes (≤2, 2-5, and ≥5 cm). From the studied tumors, 5 (8.6%) had a size of ≤2cm, 29 (50%) were between 2-5 cm and 24 (41.4%) had a size of ≥5 cm.

**Figure 1:**
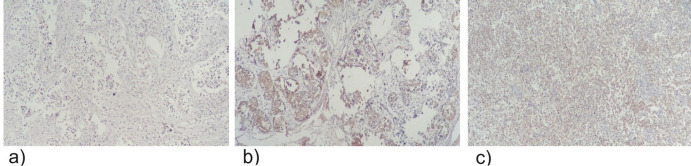
IHC results showing different p16 positivity levels: a) 1-25%, b) 26-75%, c) >75%, association between p16 positivity

After scoring the p16 positivity, it was shown that 22 (37.9%) of the patients had a score between 1-25%, 16 (27.6%) had a score of 25-75%, and 8 (13.8%) had a score of over 75% (Fig. 1). Investigation of association between P16 positivity and histologic scoring showed that of 12 patients with negative results for p16 staining, four (33.3%) had a histologic score of II, and eight (66.7%) had histologic score of III. Two (9.1%) patients from 22 with 1-25% p16 positivity, had a histologic score of I, 11(50%) had histologic score of II, and nine (40.9%) had histologic score of III. Of 16 patients with 25-75% positivity, three (18.8%) were found to have score I, five (31.2%) with score II, and eight (50%) with score III. Additionally, from the eight patients with over 75% positivity, three (37.5%) were found to have histologic score of II and five (62.5%) had a histologic score of score III. According to the Chi-square test, no significant association was found between the histologic score and p16 positivity (p=0.44). Moreover, we studied the possible association between p16 positivity and the age of the patients. Data is shown in Table 1. The results from one-way ANOVA test showed that there is no significant association between groups (p=0.67). 

**Table 1:** Frequency and relative frequency table regarding the association between p16 positivity and histologic score

**Histologic score**	**Frequency of p16 positivity**			
	**negative**	**1-25%**	**26-75%**	**>75**
I	0(0%)	2(9.1%)	3(18.8%)	0(0%)
II	4(33.3%)	11(50%)	5(31.2%)	3(37.5%)
III	8(66.7%)	9(40.9%)	8 (50%)	5(62.5%)
total	12(100%)	22 (100%)	16 (100%)	8(100%)
	$x^{2}$=5.84,df=6, p=0.44			
Mean age	52.6± 14.4	49.8±9.6	51.8±7.5	55.3±15.1
	(p=0.67)			

We also investigated the association between parameters including the size, neural, and vascular invasion with p16 positivity. Of the five tumors with diameter <2cm, one (8.3%) was negative for p16, two (9.1%) had p16 positivity of 1-25%, and two tumors (12.5%) showed positivity of 26-75%. Our results showed that from 12 patients showing no positivity to p16, one (8.3%) had a diameter of ≤2cm, five (41.7%) had a size of 2-5cm, and six (50%) were larger than 5cm. Moreover, from 23 patients with 1-25% positivity, two (9.1%) had tumors with a diameter of ≤2cm, 13(59.1%) patients had tumors ranging from 2 to 5 cm, and seven patients (31.8%) had tumors with a diameter of ≥5cm. Besides, from 16 patients with 26-75% positivity, two (12.5%) had a diameter of ≤2cm, six (37.5%) patients had tumors ranging from 2 to 5 cm, and eight (50%) patients had tumors with a diameter of ≥5cm. Of the eight patients with p16 positivity over 75%, five (62.5%) patients had tumors ranging from 2 to 5 cm, while three (37.5%) patients had tumors with a diameter of ≥5cm. According to the Chi-square test results, no significant association was found between p16 positivity and tumor size (p=0.77). 

Our results showed that from 12 patients with no positivity to p16, nine (75%) had neural invasion while no neural invasion was observed in three (25%). From 23 patients with 1-25% positivity 16 (73.7%) had neural invasion, while six (27.3%) were reported to have no neural invasion. We found that 11 (68.7%) tumors from the group with 26-75% positivity had neural invasion while five (31.3%) were reported to have no sign of neural invasion. From the group including eight patients with >75% positivity, seven (87.5%) had signs of neural invasion whereas only one (12.5%) was found without neural invasion. According to the Chi-square test results, no significant association was found between p16 positivity and neural invasion (p=0.79).

We found that 12 tumors from the group with no positivity to p16, three (25%) had perivascular invasion while no perivascular invasion was observed in the nine (75%) tumors of the group. From 23 tumors with 1-25% positivity 6 (27.3%) had perivascular invasion while 16 (72.7%) were reported to have no perivascular invasion. We found that 5(31.3%) tumors from the 16 tumors in the group with 26-75% positivity had perivascular invasion, while 11 (68.7%) were reported to have no sign of perivascular invasion. From the group including eight patients with >75% positivity, two (25%) had signs of perivascular invasion whereas six (75%) were found without perivascular invasion. The results obtained from the Chi-square test showed that there was no significant association between p16 positivity and perivascular invasion (Pearson $x^{2}$=0.17, df=3, p=0.98).

We also investigated the involvement of LNs in all studied groups. The results are represented in Table 2. According to the results of one-way ANOVA test, no significant association was found between the p16 positivity and the number of the involved LN (p=0.56). The results showed that two (16.7%) out of 12 tumors from the group with no positivity to p16 had no LN involvement, four (33.3%) had 1-3 involved LNs, three (25%) had 4-9 involved LNs, and three (25%) had more than 10 involved LNs. From 23 tumors with 1-25% positivity, four (18.2%) had no signs of LN involvement, six (27.3%) were found to have 1-3 involved LNs, two (9.1%) had 4-9 involved LNs, and 10 (45.5%) were found with ≥10 involved LNs. Moreover, of 16 patients with 26-75% positivity four (25%) had no sign of LN involvement, five (31.3%) had 1-3 involved LNs, the same number had 4-9 involved LNs, and two (12.5%) were found to have ≥10 involved LNs. From eight patients with p16 positivity of >75%, four (37.5%) were reported to have no LN involvement, two (25%) had 1-3 involved LNs, two (25%) had 4-9 involved LNs and one (12.5%) had ≥10 involved LNs. The results showed that there was no significant association between p16 positivity and the number of involved LNs (Pearson $x^{2}$=8.04, df=9, p=0.49). 

## DISCUSSION

The role of p16 in the pathology of BC has not been fully understood. Milde-Langosch et al, investigated the expression of p16 using both IHC and western blotting methods. They reported that p16 expression was significantly correlated with high BC grading. Additionally, no correlation was observed between p16 expression and clinical stage, HER2/neu, Rb expression, or Rb phosphorylation. They concluded that p16 positivity was an indicator of a more undifferentiated and malignant phenotype [15]. The results of Hashmi et al., showed that although P16 was overexpressed in their studied population with triple-negative BC, no significant association was found with recurrence of the disease [16]. 

**Table 2:** Association between p16 positivity and LN involvement

**Involved lymph nodes**	**Frequency of p16 positivity**			
	Negative	1-25%	26-75%	>75%
Number	10	18	12	5
Mean±SD (of the involved lymph nodes)	12.20±5.53	13.38±3.70	7.16±7.42	6.4±3.20
P_value_	p=0.56			
Involved lymph nodes				
0	2(16.7%)	4 (18.2%)	4(25%)	3(37.5%)
1-3	4(33.3%)	6 (27.3%)	5 (31.3%)	2 (25%)
4-9	3 (25%)	2 (9.1%)	5 (31.3%)	2 (25%)
>10	3 (25%)	10 (45.5%)	2 (12.5%)	1(12.5%)
Total	12 (100%)	22 (100%)	16 (100%)	8 (100%)
	$x^{2}$ =8.04,df=9, p=0.49			

Our investigation of histologic grade and p16 expression showed that 8 (13.8%) tumor specimens had overexpression of >75%. Golmohammadi and colleagues reported overexpression of 82% that was in association with tumor grade [17]. Our data showed that p16 expression is not associated with metastasis and involving the axillary LNs. This was inconsistent with the results of Zhao et al. They investigated the p16 in 176 BC specimens and reported that the expression was negatively associated with T grade, Bloom and Richardson score, and axillary LN metastasis (p<0.05) [18]. Wang and colleagues investigated the difference between the levels of p16 expression in BC and para-carcinoma tissues and found it significant (80.6% vs 51.6%). Moreover, they reported p16 positivity mostly in specimens with malignancy grades of I–II [12]. The P16 expression depends on the transcription factors. In this regard, while it is suppressed by YY1 and Id1, other transcription factors including CTCF, Sp1, and Ets family members induce its expression [19]. We recommend investigating the association between the expression of p16 and these transcription factors as a panel in different types of BC. 
